# Prediction of Function in ABCA4-Related Retinopathy Using Ensemble Machine Learning

**DOI:** 10.3390/jcm9082428

**Published:** 2020-07-29

**Authors:** Philipp L. Müller, Tim Treis, Alexandru Odainic, Maximilian Pfau, Philipp Herrmann, Adnan Tufail, Frank G. Holz

**Affiliations:** 1 Department of Ophthalmology, University of Bonn, 53127 Bonn, Germany; S4alodai@uni-bonn.de (A.O.); Maximilian.Pfau@ukbonn.de (M.P.); Philipp.Herrmann@ukbonn.de (P.H.); Frank.Holz@ukbonn.de (F.G.H.); 2 Center for Rare Diseases, University of Bonn, 53127 Bonn, Germany; 3 Moorfields Eye Hospital NHS Foundation Trust, London EC1V 2PD, UK; Adnan.Tufail@nhs.net; 4 Institute of Ophthalmology, University College London, London EC1V 9EL, UK; 5 BioQuant, University of Heidelberg, 69120 Heidelberg, Germany; Tim.Treis@bioquant.uni-heidelberg.de; 6 Department of Biomedical Data Science, Stanford University, Stanford, CA 94305-5464, USA

**Keywords:** retina, Stargardt disease, optical coherence tomography, visual acuity, electroretinogram, hereditary retinal disease, artificial intelligence

## Abstract

Full-field electroretinogram (ERG) and best corrected visual acuity (BCVA) measures have been shown to have prognostic value for recessive Stargardt disease (also called “*ABCA4*-related retinopathy”). These functional tests may serve as a performance-outcome-measure (PerfO) in emerging interventional clinical trials, but utility is limited by variability and patient burden. To address these limitations, an ensemble machine-learning-based approach was evaluated to differentiate patients from controls, and predict disease categories depending on ERG (‘inferred ERG’) and visual impairment (‘inferred visual impairment’) as well as BCVA values (‘inferred BCVA’) based on microstructural imaging (utilizing spectral-domain optical coherence tomography) and patient data. The accuracy for ‘inferred ERG’ and ‘inferred visual impairment’ was up to 99.53 ± 1.02%. Prediction of BCVA values (‘inferred BCVA’) achieved a precision of ±0.3LogMAR in up to 85.31% of eyes. Analysis of the permutation importance revealed that foveal status was the most important feature for BCVA prediction, while the thickness of outer nuclear layer and photoreceptor inner and outer segments as well as age of onset highly ranked for all predictions. ‘Inferred ERG’, ‘inferred visual impairment’, and ‘inferred BCVA’, herein, represent accurate estimates of differential functional effects of retinal microstructure, and offer quasi-functional parameters with the potential for a refined patient assessment, and investigation of potential future treatment effects or disease progression.

## 1. Introduction

Recessive Stargardt disease (STGD1) or *ABCA4*-related retinopathy is an important cause of inherited retinal degeneration and loss of vision in early life [1]. As an autosomal-recessive disorder, it is caused by biallelic mutations in the *ATP-binding cassette sub-family A member 4 (ABCA4*) gene (Online Mendelian Inheritance in Man # 601691) [2]. It is characterized by a generally increased fundus autofluorescence (AF) intensity followed by distinct patterns of increased and decreased AF as well as thinning of retinal layers in the optic coherence tomography (OCT) associated with loss of function [3,4,5]. However, the clinical presentation and individual progression rate are highly variable [6,7,8].

In the view of emerging interventional clinical trials, the ability to identify patients by rate of expected disease progression would be advantageous in order to maximize the potential of an intervention to demonstrate therapeutic effectiveness and improve the efficiency of future trials [7]. Several morphological and functional classifications have been proposed previously [9,10]. The full-field electroretinogram (ERG) based classification developed by Lois and colleagues has been shown to have prognostic value for disease progression and has been used in various studies to date [7,8,9,11]. However, the measurement of ERG is burdensome (i.e., time for dark adaptation), requires highly trained personnel, and is uncomfortable (e.g., due to corneal electrodes) [12]. The intensity of such tests makes patients’ cooperation problematic and in conflict with the demand for study, protocols to be time- and resource-efficient.

Biological cause and effect relationships including retinal structure–function correlations are typically multifactorial (incl. categorical parameters) and non-linear making a conventional statistical approach problematic. Recently, machine learning (ML) approaches have been introduced into ophthalmology [13,14,15]. Using algorithms that learn to perform a specific task without being explicitly programmed to use conventional logic rules, ML approaches have the potential to elucidate previously unknown correlations and have been shown to predict retinal function based on structural parameters with great accuracy providing a quasi-functional parameter (so-called ‘inferred sensitivity’) in age-related macular degeneration (AMD) [14,15]. Accurate quasi-functional parameters based on objective and easily achievable structural data might allow for simplification of patient assessments and to make study protocols less burdensome for patients without losing potentially important information.

To date, ML applications for prediction of function in *ABCA4*-related retinopathy have not been explored. We, therefore, utilized ML approaches to explore the potential to predict ERG results, the grade of visual impairment, and best corrected visual acuity (BCVA) in *ABCA4*-related retinopathy based on patients’ demographic factors and structural data of the retina. The structural retinal data were obtained using a non-invasive routinely collected OCT imaging device that is fast to obtain data. By combining multiple learning algorithms with different perspectives (ensemble ML), we aimed for more robust predictions [16]. The accuracy of the resulting ‘inferred ERG’, ‘inferred visual impairment’, and ‘inferred BCVA’ was validated in order to investigate the potential of these novel quasi-functional parameters in future patient assessment and clinical trial design.

## 2. Experimental Section

This retrospective monocenter cohort study was in adherence with the Declaration of Helsinki. The Institutional Review Board (IRB) of the University of Bonn approved the study (approval ID: #316/11 and #288/17). Written patients’ informed consent was obtained after the explanation of the nature and possible consequences of the study.

### 2.1. Subjects

The study included patients with *ABCA4*-related retinopathy based on the presence of at least one disease-causing mutation in *ABCA4* as well as the absence of mutations in *Peripherin-2* (*PRPH2)* and a phenotype compatible with STGD1 [17,18]. Genetic testing was performed as either Sanger sequencing and multiplex ligation-dependent probe amplification or next-generation sequencing. Verification of identified mutations was carried out by PCR and subsequent Sanger sequencing [19]. Patients were recruited from a clinic dedicated to rare retinal diseases. Exclusion criteria were insufficient pupil dilation, additional retinal pathology, previous vitreoretinal surgery, or other ocular comorbidities substantially affecting visual function (e.g., significant media opacity, amblyopia, or optic nerve disease). Age-matched healthy subjects without ocular disease served as controls.

### 2.2. Imaging and Functional Testing

All subjects underwent a complete ophthalmologic examination including best corrected visual acuity (BCVA) testing using Early Treatment Diabetic Retinopathy Study (ETDRS) charts, slit lamp examination, indirect ophthalmoscopy, ERG testing in accordance to standards defined by the International Society for Clinical Electrophysiology of Vision (ISCEV) using a Ganzfeld stimulator and Burian–Allan bipolar corneal electrodes (Toennies Multiliner Vision 1.70, Hochberg Germany), and standardized imaging protocol. Prior to imaging, pupils were dilated using 0.5% tropicamide and 2.5% phenylephrine. The imaging protocol consisted of AF-imaging (Spectralis HRA, Heidelberg Engineering, Heidelberg, Germany), and spectral domain (SD)-OCT (Spectralis HRA-OCT, Heidelberg Engineering, Germany). OCT was performed with single horizontal and vertical line scans centered on the fovea as well as volume scans (25° × 30°, 61 scans) with at least 20 frames per scan averaged.

**Figure 1:**
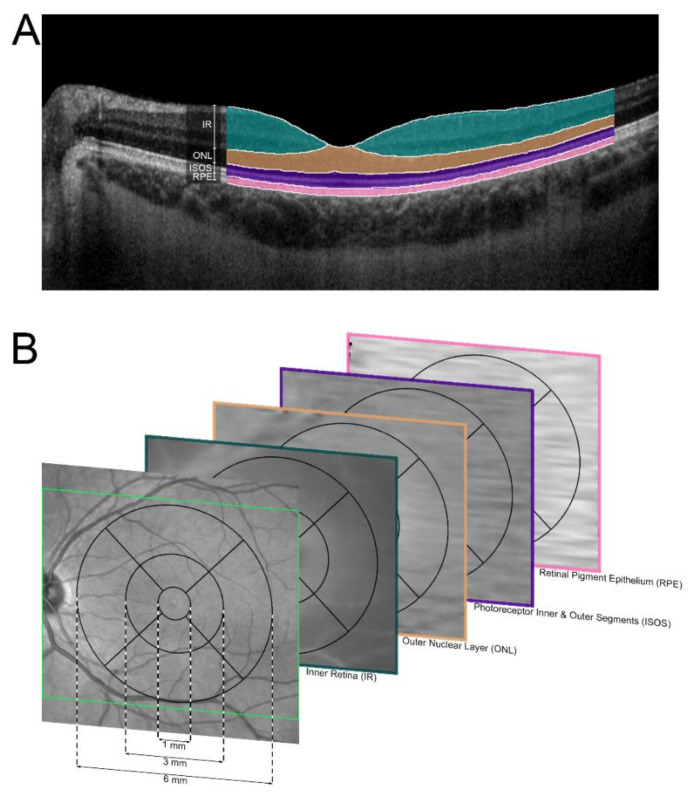
Image annotation and analysis. (**A**) Spectral-domain optical coherence tomography was semi-automatically annotated. The herein used segmentations for the inner retina (IR, green overlay), outer nuclear layer (ONL, yellow overlay), photoreceptor inner and outer segments (ISOS, purple overlay), retinal pigment epithelium (RPE, pink overlay) are highlighted color-coded. (**B**) An ETDRS grid was registered to the thickness maps of the respective OCT layers. For each subsegment, the mean thickness was extracted.

A proprietary approach for image analysis was implemented as previously described [15]. Briefly, the device-associated software (Spectralis Viewing Module 6.3.2.0, Heidelberg Engineering, Heidelberg, Germany) allowed for an initial automatic segmentation of volumetric OCT data. The segmentation was reviewed and, if indicated, manually corrected by a trained and experienced staff (A.O.). Correct Segmentation was further controlled by a senior grader (P.L.M). As the automatic segmentation tool of the device-associated software was not programed or trained for retinal pathologies associated with *ABCA4*-related retinopathy, manual correction was necessary in all patients. In cases with interruption of retinal structures (e.g., in areas of atrophy), the segmentation line was continued on the next outer segmented retinal structure. We defined the thickness between the internal limiting membrane (ILM) and the outer plexiform layer (OPL)-outer nuclear layer (ONL) boundary as ‘inner retinal (IR)’ thickness [20]. Henle fiber layer was counted towards the ‘ONL’. The photoreceptor ‘inner and outer segments (ISOS)’ ranged from band 1 (external limiting membrane, ELM) to band 3, and ‘retinal pigment epithelium (RPE)’ from band 3 to Bruch’s Membrane (BM, Figure 1) [20]. These four volumetric thickness maps for each layer were transferred as a tab-delimited file to ImageJ (U.S. National Institutes of Health, Bethesda, Maryland, USA) and overlaid with an ETDRS-Grid (i.e., circles with a diameter of 1, 3 and 6 mm, with the two eccentric rings separated into four quadrants) in order to measure the thickness of the respective layer in each of the nine subfields (Figure 1). The addition of all four retinal layers accounted for ‘full retinal (FR)’ thickness (i.e., thickness between ILM and BM).

### 2.3. Classification

Based on full-field ERG measurements, patients were categorized according to the classification developed by Lois and colleagues [9]: Group 1 contained eyes with normal scotopic and photopic responses, group 2 eyes with normal scotopic responses but reduced (over 2 standard deviations) photopic B-wave and 30-Hz flicker amplitudes, and group 3 eyes with impairment of both rod- and cone-driven responses. Using a multimodal assessment (AF and OCT), eyes were further graded according to the presence of foveal involving RPE atrophy by two independent experienced readers blinded to the results of each other, as central retinal thickness might directly be affected [21].

### 2.4. Machine Learning Analysis

Analysis was performed with Python (version 3.7.6; Python Software Foundation, Wilmington, Delaware, USA) using common data analysis packages including SciPy 1.0, Scikit-learn, XGBoost, and Eli5 [22,23,24,25]. In line with previous studies [7,26], BCVA was categorized into ‘no impairment’ (≤0.1 logMAR, ≥20/25 Snellen equivalent), ‘mild impairment’ (0.2–0.5 logMAR, 20/32–20/60), ‘moderate impairment’ (0.6–0.9 logMAR (20/80-20/160, and ‘severe impairment’ (≥1.0 logMAR, ≤20/200). Prior to modelling, categorical features were one-hot encoded (e.g., foveal status) and numerical features were normalized using a z-score transformation. For the BCVA and ERG classification as well as the BCVA regression, different ML algorithms were combined into soft voting ensembles [27,28,29]: For the prediction of the functional classifications, we used k-nearest-neighbor, random forest, support vector machine with radial basis function (rbf) kernel, and eXtreme Gradient Boosting (XGBoost) algorithm. For the functional regression, k-nearest-neighbor, kernel ridge, support vector machine with rbf kernel, and XGBoost. The algorithms were chosen based on previously demonstrated performance in the field of disease prediction through supervised learning [30,31]. To improve on their individual performance, the described sets of individual learners were combined into a stacked ensemble for more robust predictions [32]. These were then trained on three subsets of the available features: A) retinal layer thickness, B) full imaging feature set comprising retinal layer thickness and status of the fovea, and C) additional demographic features including age, refractive error (spherical equivalent), age of onset, and disease duration. The feature sets A and B included patients’ and controls’ data, while analysis with feature set C only used patients’ data as many demographic features (e.g., disease duration) was not defined for controls. We used the term ‘inferred ERG’ and ‘inferred visual impairment’ to describe the prediction of ERG and visual impairment category (classification models), and ‘inferred BCVA’ for BCVA value prediction (regression model) based on clinically feasible OCT imaging. For tuning the hyperparameters during model fitting, the F1-score (for classification) and the Root-Mean-Squared-Error (for regression) were optimized through nested cross-validation (for details see Supplementary Figure S1) as it significantly reduces bias when working with small datasets [33]. For both the inner and outer loop, a k_fold_ of 10 was chosen, resulting in an 81:9:10 split. The hyperparameters of the models used for the ensemble were optimized in the inner loop. Then, these optimized models were used to predict the holdout set of the outer loop. The importance of the used features was analyzed by employing permutation importance. Instead of removing the currently investigated feature and retraining the model, it replaces the feature with values randomly drawn from the distribution of the feature. The loss of information then indicates the importance of the relevant feature.

### 2.5. Statistical Analysis

Statistical analysis was also performed using Python (version 3.7.6; Python Software Foundation, Wilmington, Delaware, USA). For significance testing, different tests were employed depending on the nature of the tested data. For comparing two sets of data, either Welch’s t-test or Mann–Whitney U-test was used, depending on the nature of the data. For comparing multiple sets of data, the Friedmann test was used, followed up with post-hoc Nemenyi testing. A significance threshold of α = 0.05 was chosen for all tests.

## 3. Results

### 3.1. Cohort Characteristics

The study included a total of 311 eyes of 156 patients (101 female) with ABCA4-related retinopathy (mean age 38.77 years, total range 9.43–86.12 years) and 108 eyes of 54 controls (35 female, mean age 39.24 years, total range 13.14–77.02 years). One eye of one patient was excluded due to status post retinal detachment. The distributions of age at baseline and sex did not differ between patients and controls (Table 1 and Supplementary Figure S2). Individual demographic and genetic data can be found in Supplementary Table S1.

Based on the full-field ERG classification [9], 71 patients were assigned to group 1, 55 patients to group 2, and 30 patients to group 3. Both eyes of an individual patient always met the same group classification. The classification closely correlated with the phenotypes [4,8,10]: Group 1 eyes typically revealed localized foveal alterations surrounded by single flecks and a homogeneous background, group 2 eyes showed atrophic changes at the posterior pole with a more heterogeneous background caused by numerous flecks and group 3 eyes were associated multiple widespread atrophic areas at the macula with a heterogeneous background. The distribution of sex between the groups was similar. Group 2 patients revealed the highest age at exam and age of onset, while group 1 and 3 patients had the shortest and longest disease duration, respectively. Group 3 eyes were more myopic compared to eyes assigned to both other groups and showed foveal non-involvement of RPE atrophy much less frequently. The latter was in line with the significantly worse visual acuity in group 3 eyes compared to group 1 and 2 eyes (Table 1 and Supplementary Figure S2).

### 3.2. Retinal Layer Thickness

Overall, patients with ABCA4-related retinopathy revealed reduced macular full retinal thickness compared to controls (whole ETDRS grid [mean ± standard deviation]: patients, 247.92 ± 36.18 µm; controls, 305.63 ± 13.81 µm; *p* < 0.001; Supplementary Figure S3). This difference mainly derived from a thinner ONL (patients, 33.46 ± 13.83 µm; controls, 61.88 ± 7.09 µm; *p* < 0.001). ISOS (patients, 30.48 ± 13.60 µm; controls, 44.15 ± 11.30 µm; *p* < 0.001) and IR (patients, 149.67 ± 14.37 µm; controls, 163.63 ± 11.30 µm; *p* < 0.001) also accounted for the thinning, while RPE (patients, 34.31 ± 5.63 µm; controls, 35.97 ± 2.18 µm; *p* = 0.089) did not show significant differences between patients and controls.

**Figure 2:**
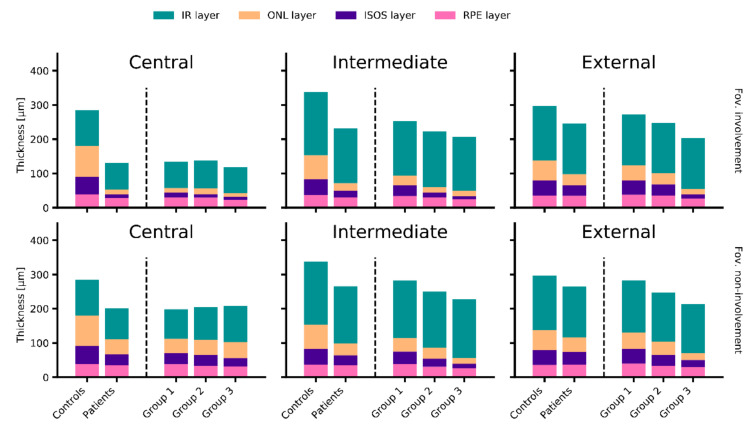
Retinal layer thickness measurements, eccentricity and foveal status. The bar plots demonstrate the mean retinal layer thickness measurements in the central subfield (first column), intermediate subfields (second column) and external subfields (third column) of the ETDRS, for controls’ and patients’ eyes depending on the presence (first row) or absence (second row) of foveal involvement. Patients’ eyes were further divided according to full-field electroretinogram based group classifications. Compared to controls, patients’ eyes revealed reduced retinal thickness in each eccentricity, especially in the outer nuclear layer (ONL, yellow) and the photoreceptor inner and outer segment layers (ISOS, purple). In the central subfield, the retinal thinning was expressed in eyes assigned to all groups of patients, while in the more eccentrically subfields, it was increasingly expressed from group 1 over group 2 to group 3 eyes. Concerning the foveal status, the most obvious difference was noticeable in the central subfield, where the retinal thinning was distinctly more pronounced in eyes with foveal involvement. The bar plots and exact numbers of measurements according to each ETDRS subfield can be found in Supplementary Figure S3 and in Supplementary Table S2, respectively. IR: inner retina (green), RPE: retinal pigment epithelium (pink).

The differences in retinal thickness between patients and controls were most pronounced in the central ETDRS subfield (FR: patients, 156.10 ± 55.04 µm; controls, 285.11 ± 25.05 µm; *p* < 0.001), but still significant in the intermediate (FR: patients, 243.23 ± 40.55 µm; controls, 338.01 ± 17.98 µm; *p* < 0.001) and external (FR: patients, 252,71 ± 37.48 µm; controls, 296.80 ± 14.46 µm; *p* < 0.001) ETDRS subfields (Figure 2 and Supplementary Figure S3). The central retinal thinning was distinctly less expressed in eyes with foveal non-involvement (FR: foveal involvement, 131.28 ± 36.19 µm; foveal non-involvement, 201.67 ± 54.57 µm; *p* < 0.001), while the foveal status was of minor importance in the intermediate (FR: foveal involvement, 230.99 ± 33.99 µm; foveal non-involvement, 265.70 ± 41.98 µm; *p* < 0.001) and external (FR: foveal involvement, 246.24 ± 37.94 µm; foveal non-involvement, 264.58 ± 33.53 µm; *p* < 0.001) ETDRS subfields (Figure 2).

Comparisons between the three ERG groups revealed that group 3 eyes showed the thinnest retina in all analyzed eccentricities (*p* < 0.001) with measures of 130.59 ± 44.65 µm, 209.89 ± 29.95 µm and 204.76 ± 27.38 µm for full retina in the central, intermediate and external ETDRS subfields. While group 1 and 2 revealed similar retinal thickness in the central subfield (full retina: 161.13 ± 53.33 µm and 163.50 ± 53.33 µm for group 1 and 2, respectively; *p* = 0.426), there was a significant difference in the intermediate (full retina: 265.31 ± 33.86 µm and 232.91 ± 36.78 µm; *p* < 0.001) and external ETDRS subfields (full retina: 276.76 ± 20.02 µm and 247.81 ± 32.15 µm; *p* < 0.001; Figure 2 and Supplementary Figure S3).

In order to evaluate potential directional preferences of disease manifestation, we analyzed the retinal thinning relative to controls in the superior, inferior, nasal, and temporal subfields, which was overall significantly different (*p* < 0.001): The nasal ETDRS subfields revealed the highest and the superior subfields the lowest difference between patients and controls, respectively (Supplementary Figure S4).

Further layer thickness measurements for each ETDRS subfield can be found in Supplementary Figure S3 and Supplementary Table S2.

### 3.3. Prediction of Panretinal Function

**Figure 3:**
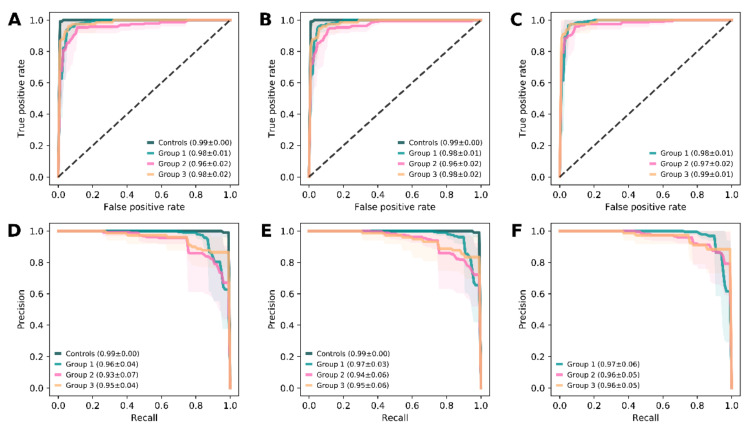
Model performance for the prediction of panretinal function. Receiver operating characteristic (ROC) curves that describe the true positive rate versus the false positive rate (top row, **A–C**), and graphs that plot the precision against the recall (bottom row, **D–F**) are demonstrated for the models predicting panretinal function based on feature set **A** (first column, **A** and **D**), **B** (second column, **B** and **E**), and **C** (third column, **C** and **F**). The lines represent the mean (± standard deviation, shaded area) of all folds for each group category according to full-field electroretinogram results, and the given values are the respective area under the curve (mean ± standard deviation). The plots and respective values demonstrate the high robustness and power of the models to predict the panretinal function. The use of pure retinal thickness data (feature set **A**, first column) already reached the performance level of the models using additional features (feature set **B**, second column; feature set **C**, third column).

Using our classifier ensemble model, panretinal function in terms of full-field ERG results (groups 1 to 3, as proposed by Lois and colleagues) [9] could accurately be predicted based on each feature set without much difference: With feature set A (i.e., pure objective retinal layer thickness data), the accuracy of the prediction was 99.68 ± 0.64% to differentiate controls and patients, as well as 94.63 ± 2.57%, 93.52 ± 2.49%, and 96.99 ± 2.51% to categorize patients into groups 1, 2, and 3, respectively (Figure 3A,D). Using additional features did not lead to significantly more accuracy in this context: For feature set B (i.e., all structural data), the model correctly differentiated controls from patients with 99.53 ± 1.02% accuracy, and patients into groups 1, 2 and 3 with accuracies of 93.84 ± 2.18%, 93.36 ± 2.22% and 96.84 ± 2.36%, respectively (Figure 3B,E). For feature set C (i.e., structural and demographic data), the accuracy of the prediction revealed values of 94.79 ± 1.86% (group 1), 95.11 ± 1.92% (group 2) and 97.47 ± 2.03% (group 3, Figure 3C,F).

The weights that the model has assigned to each feature was again assessed using permutation importance. For pure retinal thickness features (feature set A; Supplementary Figure S5A) as well as all imaging features (feature set B; Supplementary Figure S5B), the thickness of the ONL, ISOS, and IR had distinctly more impact for the correct prediction of panretinal function than RPE and foveal status. All three eccentricities (Central, intermediate, and external) were represented among the most important features. Including additional demographic features (feature set C; Supplementary Figure S5C), age of onset and refractive error/spherical equivalent also showed high relevance, while age at exam and disease duration ranked within the second quarter of important factors for the model.

### 3.4. Prediction of Visual Impairment

**Figure 4:**
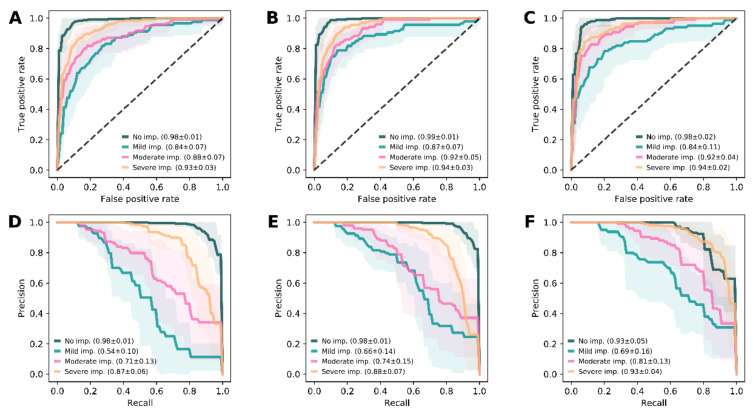
Model performance for the prediction of visual impairment. Receiver operating characteristic (ROC) curves that describe the true positive rate versus the false positive rate (top row, **A–C**), and graphs that plot the precision against the recall (bottom row, **D–F**), are demonstrated for the models predicting the grade of visual impairment (imp.) based on feature set **A** (first column, **A** and **D**), **B** (second column, **B** and **E**), and **C** (third column, **C** and **F**). The lines represent the mean (± standard deviation, shaded area) of all folds for category according to visual impairment, and the given values are the respective area under the curve (mean ± standard deviation). The robustness and power of the models to predict the grade of visual impairment was best for correct prediction of the extreme values (no impairment, dark green; severe impairment, yellow). The use of pure retinal thickness data (feature set **A**, first column) already reached the performance level of the models using additional features (feature set **B**, second column; feature set **C**, third column).

Similar to panretinal function, the classifier ensemble models were trained to predict the grades of visual impairment: Using features set A, prediction of no, mild, moderate and severe visual impairment reached accuracies of 92.25 ± 1.52%, 86.10 ± 3.78%, 89.27 ± 5.65%, and 88.64 ± 4.01%, respectively (Figure 4A,D). With features set B, prediction showed accuracies at the same level with values of 93.68 ± 2.25%, 89.10 ± 3.27%, 90.23 ± 5.62%, and 88.17 ± 4.39%, respectively (Figure 4B,E). The same was true for feature set C, where the accuracies of the predictions were 91.44 ± 3.42%, 86.84 ± 3.75%, 89.57 ± 3.83%, and 87.26 ± 3.91%, respectively (Figure 4C,F).

Analysis of the individual structural feature importance (feature set A and B) revealed that the foveal status was the most important feature for predictions, followed by layer thickness measures (especially, ONL, ISOS, and IR) in the central ETDRS subfield. Measures in the intermediate and external ETDRS subfields were of less impact. For feature set C, all demographic parameters were within the first twelve ranks but did not reach the feature importance of foveal status (Supplementary Figure S5D,F).

### 3.5. Prediction of Best Corrected Visual Acuity

**Figure 5:**
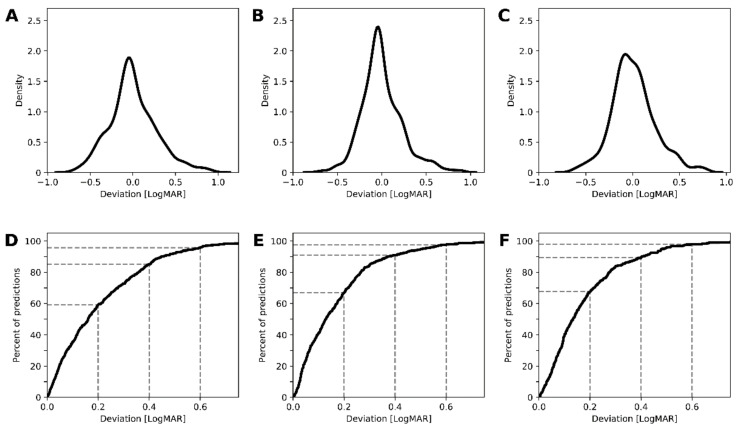
Model performance for the prediction of best corrected visual acuity (BCVA). The density plots that demonstrate the deviation of the predictions from the measured BCVA values (top row, **A–C**), and graphs that plot the cumulative true positive rate against the deviation (bottom row, **D–F**), are demonstrated for the models based on feature set **A** (first column, **A** and **D**), **B** (second column, **B** and **E**), and **C** (third column, **C** and **F**). The inclusion of foveal status with (feature set **C**, third column) or without (feature set **B**, second column) demographic data improved the performance of the ensemble machine learning algorithms compared to pure retinal thickness data (feature set **A**, first column). LogMAR: Logarithm of the Minimum Angle of Resolution.

Apart from the grades of visual impairment, the actual visual acuity values were predicted with ensemble ML algorithms. Compared to the pure retinal layer thickness model (feature set A, Figure 5A,D), inclusion of foveal status resulted in significantly improved accuracy of the prediction as shown by values for feature set B (Figure 5B,E) or feature set C (Figure 5C,F): for example, the percentage of BCVA prediction within ±0.3 LogMAR (15 Letters, three lines on the LogMAR chart) of the ground truth was 72.67%, 85.31%, and 83.92% for the feature sets A, B and C, respectively (Table 2). In this context, the feature set B model revealed even less deviation from the ground truth compared to feature set C, which could be explained by the inclusion of controls with uniform good visual acuity in feature sets A and B. Irrespective of the model, the deviation between prediction and true value increased towards the extreme BCVA values (Supplementary Figure S6).

Analysis of the feature permutation importance values revealed that for prediction of BCVA, foveal status carried the most weight. This was followed by OCT layer thickness measures in the central ETDRS subfield, especially for ONL and ISOS. Including demographic factors, age of onset and disease duration ranked at the level of RPE and IR measures in the central ETDRS subfield (i.e., behind foveal status and ONL and ISOS in the central subfield). Age at exam was of medium relevance and spherical equivalent carried very low importance (Supplementary Figure S5G–I).

## 4. Discussion

Based on a distinct structure-function correlation between OCT layer thickness and panretinal (ERG) as well as central retinal (BCVA) function, the possibility to predict retinal function in *ABCA4*-related retinopathy by imaging data and patients’ demographic parameters was demonstrated by this study. These predictions of inferred functional categories (‘inferred ERG’ and ‘inferred visual impairment’) obtained by ensemble ML algorithms reached an accuracy of up to 97.47 ± 2.03% and 93.68 ± 2.25%, respectively. The pure objective data from retinal layer thickness measures (feature set A) already achieved these levels of exactness. Including further simply determinable features (feature set B and C, especially foveal status) into the models, absolute BCVA values could also be predicted (‘inferred BCVA’) with high precision (Table 2). Apart from substantiating the functional relevance of structural alterations in *ABCA4*-related retinopathy, these novel calculated parameters may serve as quasi-functional surrogate markers in patient assessment and future clinical trials.

The term ‘ML’ covers different approaches to artificial intelligence that have been targeted on computers to learn without being explicitly programmed since 1959 [34]. In the last decade, ML techniques have entered visual science, including analysis of retinal images [13]. Due to demographic changes, the number of affected individuals with retinopathies, including diabetic retinopathy and AMD, has been increasing. The ML techniques were, therefore, mostly used for automatic imaging analysis in order to detect and classify pathologic features, where the power of conventional logic rules and linear approaches is quite restricted [35]. More recently, ML approaches have been used to predict retinal function based on structural parameters similar to the inferred retinal function ML model presented in this study that has shown to provide a highly sensitive tool. Rohm and colleagues predicted future course of visual acuity in treated neovascular AMD by database parameters and OCT data achieving comparable result to VA measurement reliability for three months [14]. Also, for neovascular AMD, von der Emde and colleagues achieved high accuracy by using ML analysis of OCT data to estimate fundus controlled perimetry results (also called ‘inferred sensitivity’), giving a quasi-functional surrogate marker and an additional spatial dimension [15]. To date, only one publication showed the possible application of artificial intelligence in *ABCA4*-related retinopathy showing automatic cone photoreceptor localization for adaptive optics imaging in Stargardt disease, achromatopsia, and retinitis pigmentosa [36]. Previous studies for *ABCA4*-related retinopathy that investigated the evidence for structure–function correlations between retinal sensitivity and multimodal imaging only used a limited number of narrowly selected predictors and/or application of linear models [37,38]. As biological relationships including retinal structure–function correlations are typically multifactorial (including categorical parameters such as foveal status) and non-linear, correlations found in these studies have to be interpreted carefully. Especially categorical predictions (i.e., ERG-based groups or grade of visual impairment) might be difficult to assess (manual thresholds for each included factor). In contrast, our model was able to identify relationships between variables without manual intervention. Using an ensemble ML approach, different statistical perspectives were combined to increase the robustness of the demography- and morphology-based functional prediction (inferred retinal function) in *ABCA4*-related retinopathy [16]. Furthermore, this approach further enabled us to evaluate the effect and importance of individual predicting parameters.

The fact that (apart from foveal status) ONL, which includes light-sensitive photoreceptor cells bodies, and the ISOS, which includes the photoreceptor inner and outer segments (i.e., the location of phototransduction), consistently were among most important imaging-based predictors of retinal function in our model is biologically plausibile. In a previous study of AMD, ONL exhibited the highest feature importance for inferred sensitivity [39]. Importance of features of the model may not necessarily indicate a biological correlation. Of note, IR was significantly thicker than other layers and, therefore, less prone to grading errors. Together with the slight but uniform thinning of this layer, it possibly explains the relatively high feature importance of IR. The correlation of IR thinning with measures of the outer retinal layers might suggest some form of functional syncytium through the whole retina. This could explain previously described inner retinal changes in *ABCA4*-related retinopathy and retinal remodeling processes that accompany photoreceptor loss [40]. Further evidence of the biological plausibility of our model is given by the differential effect of location on central retinal and panretinal function: While the imaging feature importance ranking for BCVA prediction (category and value) was dominated by measurements within the central ETDRS subfield that covers the central 3° of the retina, measurements in the intermediate and external ETDRS subfields had more weight in predicting ERG results (i.e., panretinal function). The distribution of demographic factors between the different ERG-based groups explained their relatively high feature importance: for example, group 3 eyes had earliest age of onset, most negative median refractive error (spherical equivalent), and the longest disease duration (Table 1). Interestingly, a tendency towards negative refractive errors has been previously reported in the setting of *ABCA4*-related retinopathy [41].

Optimizing clinical trial design, including the selection of suitable patients and endpoints, constitutes a prerequisite towards an accurate assessment of potential therapeutic effects. Several approaches have been brought forward to predict progression in patients with *ABCA4*-related retinopathy. Some of those use genetic information, others rely on various imaging parameters [6,17]. The full-field electroretinogram (ERG) based classification developed by Lois and colleagues was shown to have a distinct prognostic value for disease progression and has, therefore, been used by several recent clinical studies [7,9,11]. However, the measurement of ERG is often described as uncomfortable and burdensome (i.e., time for dark adaptation), which is problematic when delivering clinical studies and time-efficient patient appointments [12]. We demonstrated the possibility to infer retinal function based on routinely obtained structural imaging and patients’ demographic data using ensemble ML algorithms with high safety levels. The use of ‘inferred BCVA’, ‘inferred visual impairment’, and ‘inferred ERG’ as quasi-functional surrogate markers and endpoints could provide distinct advantages over the respective conventional functional measurements: (I) OCT has become a ubiquitously available imaging device and so commonly used to assess morphologic outcome measure in both routine examination and clinical trials [42]. Therefore, the utility of inferred retinal function could facilitate more time-efficient research studies, while offering comparable benefits to direct functional testing, including prediction of disease progression and objective differential testing of cone and rod function. (II) Digital imaging offers a higher spatial resolution and less variability than psychophysical testing. (III) The application of our models allows for simultaneous calculation of ‘inferred BCVA’, ‘inferred visual impairment’ and ‘inferred ERG’. (IV) Structural data are faster to obtain and even applicable in patient unsuitable for psychophysical testing. (V) ‘Inferred BCVA’, ‘inferred visual impairment’ and ‘inferred ERG’ could be compared across diseases to potentially facilitate objective cost–benefit analysis. Giving these advantages, patient assessment, selection and study visits might become more time- and resource-effective by using inferred retinal function.

The International Conference on Harmonization Guidelines on Statistical Principles for Clinical Trials has established that the “evidence for surrogacy depends upon (I) the biological plausibility of the relationship, (II) the demonstration in epidemiologic studies of the prognostic value of the surrogate for the clinical outcome, and (III) evidence from clinical trials that treatment effects on the surrogate correspond to effects on the clinical outcome” [43]. The biological plausibility of the inferred retinal function has been shown as stated above. In contrast to conventional surrogate markers that are not directly related to function, ‘inferred BCVA’, ‘inferred visual impairment’, and ‘inferred ERG’ represent quasi-functional endpoints minimizing the relevance of the second and third criterion. Further, the use of nested cross-validation underlines the high accuracy of the model for unknown data. Nevertheless, ML models are primarily limited by the training data, which is the basis of the predictions. Other disease entities or atypical clinical presentations (as possible in interventional clinical trials) would necessitate further training data in order to define the appropriate applicability domain.

Of note, the complexity of the ensemble ML approach might theoretically require a slightly increased number of training examples compared to simple (i.e., non-ensemble) artificial intelligence approaches. Nevertheless, our results using ensemble ML algorithms revealed enhanced prediction accuracy for inferred function compared to published simple ML models for retinal diseases [14]. High accuracy is a prerequisite for future application of this quasi-functional surrogate marker. Apart from the utilization of a sophisticated ML model, the enhanced prediction accuracy in our cohort might be accounted for by looking at a simple monogenetic disease entity together with strict inclusion, as well as exclusion criteria in this study. We excluded other retinal diseases or comorbidities that could substantially affecting visual function including optic nerve diseases and cataract. This might result in a limitation of our study as additional retinal pathologies with functional relevance (e.g., changes in the inner retinal layers due to glaucoma) could be underrepresented in our model. However, clinical trials usually use as least as stringent inclusion criteria in order to generate a uniform study cohort, indicating that the restrictive inclusion and exclusion criteria might only be a theoretical limitation. Similar to recent studies (e.g., multicenter PROGSTAR study) [17,18], the diagnosis of *ABCA4*-related retinopathy was based on a compatible phenotype and the presence of at least one disease-causing mutation in *ABCA4* as well as the absence of mutations in *Peripherin-2* (*PRPH2)*. Even if a more stringent definition (i.e., only patients with proven biallelic pathogenic mutation) would be more certain, the commonly accepted and here-used definition of *ABCA4*-related retinopathy is currently the best tradeoff between cohort size and limitations of genetic testing [44]. By using mean retinal layer thickness within the ETDRS subfields, focal alterations might not be detected and the predictive value it holds might not be attributed. For example, local thinning and thickening of RPE (due to flecks) might be balanced within the subfield. However, as group 3 eyes (that also showed most impaired BCVA) revealed consistently reduced RPE (Supplementary Table S2), this specific retinal layer, nevertheless, was attributed distinct feature importance (Supplementary Figure S5). A parameter that was not considered in the study was the area of peripapillary sparing, which can be assessed quantitatively and has been shown to correlate with electroretinographic findings in this disease entity [45,46]. Future models for ‘inferred ERG’ might include this parameter to further improve the predictions. To safely utilize such an ensemble ML model in future studies, the longitudinal accuracy of the model on the respective study cohort needs to be tested on a subset of subjects prior to inferring the functional data for the remaining participants. It has been established that BCVA (as well as ‘inferred BCVA’) does not constitute an optimal endpoint in *ABCA4*-related retinopathy due to its high interindividual variability and the phenomenon of foveal non-involvement [8]. The future evaluation of ‘inferred sensitivity’ based on fundus-controlled perimetry (as described for AMD [15]) might, therefore, be warranted as the high-resolution mapping of localized functional impairment might overcome this limitation and provide an additional dimension [47]. As this specific AMD study was also restricted to a simple ML model, the application of our ensemble ML approach (i.e., using different statistical perspectives) might also allow for improved accuracy in this context.

In conclusion, a consistent structure–function relationship in ABCA4-related retinopathy could be demonstrated. Based on this correlation, we have introduced the artificial intelligence-based analysis strategy of ‘inferred BCVA’, ‘inferred visual impairment’, and ‘inferred ERG’ to estimate differential effects of retinal structural alterations on central retinal and panretinal as well as cone and rod function in ABCA4-related retinopathy. This method potentially constitutes a valuable tool to predict retinal function without the need of psychophysical examinations enabling fast routine patients’ assessment and efficient study protocols without loss of information. The conception of ‘inferred BCVA’, ‘inferred visual impairment’, and ‘inferred ERG’ as quasi-functional outcome measures are not restricted to ABCA4-related retinopathy but might be further applied to other retinal diseases.

## Figures and Tables

**Table 1:** Demographic and functional data of all included subject.

Patient-Level Data	Controls	All Patients	Group 1	Group 2	Group 3
Patients (*n*)	54	156	71	55	30
Sex (F/M)	35/19	101/55	46/25	36/19	19/11
Age at Exam (Y)^A^	39.24 ± 16.00	38.77 ± 18.23	33.76 ± 15.17	45.03 ± 19.521	39.15 ± 18.79
Age of Onset (Y)^A^	---	26.39 ± 17.16	24.75 ± 15.48	32.91 ± 18.93	18.30 ± 12.59
Disease Duration (Y)^A^	---	12.39 ± 12.45	9.01 ± 9.55	12.12 ± 12.00	20.85 ± 15.07
Eye-Level Data					
Eyes (*n*)	108	311	142	109	60
BCVA (LogMAR)^A^	0.00 ± 0.00	0.68 ± 0.47	0.57 ± 0.44	0.62 ± 0.43	1.06 ± 0.4
Refractive Error (Dpt)^A^	−0.80 ± 2.05	−1.15 ± 1.99	−1.23 ± 2.05	−0.64 ± 1.95	−1.88 ± 1.67
Foveal Status (FI/FNI)	---	197/114	79/63	65/44	53/7

^A^ Values indicate mean ± standard deviation. BCVA: best corrected visual acuity, dpt: diopters, f: female, FI: foveal involvement, FNI: foveal non-involvement, LogMAR: logarithm of the minimum angle of resolution, M: Male, Y: Years.

**Table 2:** Accuracy of predicting best corrected visual acuity (BCVA).

Maximal Deviation from Truth [LogMAR]	Feature Sets [%] ^A^		
	A	B	C
−0.5 To 0.5	92.10	96.37	95.82
−0.4 To 0.4	85.15	92.42	89.35
−0.3 To 0.3	72.67	85.31	83.92
−0.2 To 0.2	59.24	75.36	67.64
−0.1 To 0.1	35.39	53.55	39.87

^A^ Values indicate the percentages of predicted BCVA values that fall within the interval of deviation from the truth. LogMAR: logarithm of the minimum angle of resolution.

## Supplementary Materials

The following are available online at https://www.mdpi.com/2077-0383/9/8/2428/s1, Supplementary Figure S1: Visualization of the nested cross-validation, Supplementary Figure S2: Cohort characteristics of included participants, Supplementary Figure S3: Retinal layer thickness measurements, Supplementary Figure S4: Directional differences in retinal thickness, Supplementary Figure: Feature importance for the machine learning algorithms, Supplementary Figure S6: Deviation of the best corrected visual acuity (BCVA) predictions, Supplementary Table S1: Individual demographic and genetic data of included patients with ABCA4-related retinopathy, and Supplementary Table S2: Retinal layer thickness measures for each layer and subfield.
