# Supplementary material for: Prediction of Function in ABCA4-Related Retinopathy Using Ensemble Machine Learning
Source: J Clin Med. 2020 Jul 29;9(8):2428. doi: 10.3390/jcm9082428 (PMC7463567; doi:10.3390/jcm9082428)
Supplement: Supplementary file 1 [file jcm-09-02428-s001.zip › Supplementary_Table S1 New.pdf]

Supplementary Table S1: Individual demographic and genetic data of included patients with *ABCA4*-related retinopathy

Supplement to *Prediction of function in ABCA4-related retinopathy using ensemble machine learning*

Group	#	Sex	Age [yrs]	Age of Onset [yrs]	*ABCA4*-Mutation Allele 1	*ABCA4*-Mutation Allele 2
1	1	m	11	10	c.1622T>C / p.(Leu541Pro)<br>c.3113C>T / p.(Ala1038Val)	---
1	2	m	44	44	c.5603A>T / p.(Asn1868Ile)<br>c.6148G>C / p.(Val2050Leu)	c.454C>T / p.(Arg152*)
1	3	f	13	16	c.5882G>A / p.(Gly1961Glu)	c.5917delG/ p.(Val1973*)
1	4	f	66	67	c.768G>T / p.(Val256Val)	c.5603A>T / p.(Asn1868Ile)
1	5	f	52	53	c.5461-10T>C<br>c.5603A>T / p.(Asn1868Ile)	c.5603A>T / p.(Asn1868Ile)
1	6	f	20	54	c.5882G>A / p.(Gly1961Glu)	c.5916+2T>C (SSM)
1	7	f	27	33	c.2588G>C / p.(Gly863Ala)<br>c.5603A>T / p.(Asn1868Ile)	c.6449G>A / p.(Cys2150Tyr)
1	8	m	17	17	c.1622T>C / p.(Leu541Pro)<br>c.3113C>T / p.(Ala1038Val)	c.2588G>C / p.(Gly863Ala)<br>c.5603A>T / p.(Asn1868Ile)
1	9	f	19	30	c.206G>A / p.(Trp69*)	c.5882G>A / p.(Gly1961Glu)
1	10	f	50	51	c.3322C>T / p.(Arg1108Cys)	c.5882G>A / p.(Gly1961Glu)
1	11	f	13	19	c.45G>A / p.(Trp15*)	c.5882G>A / p.(Gly1961Glu)
1	12	f	59	59	E20-E22 Deletion	c.5603A>T / p.(Asn1868Ile)
1	13	m	7	21	c.1253T>C / p.(Phe418Ser)<br>c.656G>C / p.(Arg219Thr)	c.2588G>C / p.(Gly863Ala)<br>c.5603A>T / p.(Asn1868Ile)
1	14	f	18	41	c.768G>T / p.(Val256Val)	c.5882G>A / p.(Gly1961Glu)
1	15	f	43	45	c.634C>T / p.(Arg212Cys)<br>c.2588G>C / p.(Gly863Ala)	c.5603A>T / p.(Asn1868Ile)
1	16	f	25	27	c.194G>A / p.(Gly65Glu)	c.5603A>T / p.(Asn1868Ile)
1	17	f	20	37	c.5882G>A / p.(Gly1961Glu)	c.5916+2T>C (SSM)
1	18	m	32	34	E6 c.629T>A (p.(Leu210Gln)	c.4328G>A / p.(Arg1443His)
1	19	m	10	17	c.956T>G / p.(Leu319Arg)	c.6449G>A / p.(Cys2150Tyr)
1	20	f	28	38	c.5762_5763dupTG /<br>p.(Ala1922Trpfs*18)	c.5882G>A / p.(Gly1961Glu)
1	21	m	51	75	c.5603A>T / p.(Asn1868Ile)	c.3210_3211dupGT /<br>p.(Ser1071Cysfs*14)<br>c.5603A>T / p.(Asn1868Ile)
1	22	f	50	61	c.5189G>A / p.(Trp1730*)	c.5603A>T / p.(Asn1868Ile)
1	23	f	39	43	c.5882G>A / p.(Gly1961Glu)	----
1	24	m	36	37	c.1622T>C / p.(Leu541Pro)<br>c.3113C>T / p.(Ala1038Val)	c.6089G>A / p.(Arg2030Gln)
1	25	f	32	47	c.3813+9G>C<br>c.3819dupT / p.(Leu1274Serfs*8)	c.5882G>A / p.(Gly1961Glu)

1	26	f	33	36	c.2588G>C / p.(Gly863Ala)<br>c.5603A>T / p.(Asn1868Ile)	c.3871C>T / p.(Gln1291*)
1	27	m	5	18	E17 c.2588G>C / p.(Gly863Ala)<br>c.5603A>T / p.(Asn1868Ile)	c.5461-10T>C<br>c.5603A>T / p.(Asn1868Ile)
1	28	f	12	13	c.1957C>T / p.(Arg653Cys)	c.5882G>A / p.(Gly1961Glu)
1	29	m	14	41	c.1584C>A / p.(Tyr528*)	c.5882G>A / p.(Gly1961Glu)
1	30	m	42	58	c.4918C>T / p.(Arg1640Trp)	c.5693G>A / p.(Arg1898His)
1	31	w	11	12	c.2041C>T / p.(Arg681*)	c.6079C>T / p.(Leu2027Phe)
1	32	w	6	16	c.1309delC / p.(Gln437Argfs*12)	c.5882G>A / p.(Gly1961Glu)
1	33	m	5	21	c.5461-10T>C<br>c.5603A>T / p.(Asn1868Ile)	c.5882G>A / p.(Gly1961Glu)
1	34	f	43	44	c.634C>T / p.(Arg212Cys)	c.3292C>T / p.(Arg1098Cys)
1	35	m	8	14	c.694C>T / p.(Cys232Phe)<br>c.2626C>T / p.(Gln876*)	c.5882G>A / p.(Gly1961Glu)
1	36	f	25	29	c.1822T>A / p.(Phe608Ile)	c.5603A>T / p.(Asn1868Ile)
1	37	f	30	37	c.4463G>A / p.(Cys1488Tyr)<br>c.6148G>C / p.(Val2050Leu)	c.5882G>A / p.(Gly1961Glu)
1	38	f	6	27	c.872C>T / p.(Pro291Leu)<br>c.5603A>T / p.(Asn1868Ile)	c.6310dupC / p.(Gln2104Profs*31)
1	39	f	27	35	c.2300T>A / p.(Val767Asp)	c.5882G>A / p.(Gly1961Glu)
1	40	m	47	58	c.3871C>T / p.(Gln1291*)	c.5882G>A / p.(Gly1961Glu)
1	41	f	27	29	c.5882G>A / p.(Gly1961Glu)	c.5917delG / p.(Val1973*)
1	42	m	5	36	c.2588G>C / p.(Gly863Ala)<br>c.5603A>T / p.(Asn1868Ile)	c.3085C>T / p.(Gln1029*)
1	43	f	18	30	c.1622T>C / p.(Leu541Pro) ;<br>c.3113C>T / p.(Ala1038Val)	c.5603A>T / p.(Asn1868Ile)
1	44	f	6	15	c.194G>A / p.(Gly65Glu)	c.2588G>C / p.(Gly863Ala)
1	45	f	30	40	c.4918C>T / p.(Arg1640Trp)	---
1	46	f	30	36	c.5512C>G / p.(His1838Asp)<br>c.5882G<A / p.(Gly1961Gln)	c.5882G<A / p.(Gly1961Glu)
1	47	m	14	15	c.3813G>C / p.(Glu1271Asp)	---
1	48	m	10	10	c.3813G>C / p.(Glu1271Asp)	---
1	49	f	17	24	c.1309delC / p.(Gln437Argfs*12)	c.5882G>A / p.(Gly1961Glu)
1	50	f	12	12	c.4793C>A / p.(Ala1598Asp)	c.4793C>A / p.(Ala1598Asp)
1	51	m	6	22	c.634C>T / p.(Arg212Cys)	c.5882G<A / p.(Gly1961Gln)
1	52	f	29	50	c.634C>T / p.(Arg212Cys)	c.2588G>C / p.(Gly863Ala)<br>c.5603A>T / p.(Asn1868Ile)
1	53	f	36	37	c.2712del / p.(Glu905Argfs*27)	c.5603A>T / p.(Asn1868Ile)
1	54	m	24	25	c.5603A>T / p.(Asn1868Ile)	c.6419T>G / p.(Leu2140Arg)
1	55	f	52	56	c214G>A / p.(Gly72Arg)	c.5603A>T / p.(Asn1868Ile)

1	56	f	6	10	c.1622T>C / p.(Leu541Pro)<br>c.3113C>T / p.(Ala1038Val)	E13 Deletion
1	57	f	39	40	c.1622T>C / p.(Leu541Pro)<br>c.3113C>T / p.(Ala1038Val)	c.6089G>A / p.(Arg2030Gln)
1	58	f	10	24	c.1622T>C / p.(Leu541Pro)<br>c.3113C>T / p.(Ala1038Val)	c.3375_3377delTCC /<br>p.(1125_1126del)
1	59	m	24	32	E6 c.716G>A / p.(Trp239*)	c.5882G>A / p.(Gly1961Glu)
1	60	m	14	22	c.5882G>A / p.(Gly1961Glu)	c.6729+19delGTTGGCCCTGGGG
1	61	m	15	31	c.2588G>C / p.(Gly863Ala)<br>c.5603A>T / p.(Asn1868Ile)	c.6449G>A / p.(Cys2150Tyr)
1	62	f	33	37	c.3113C>T / p.(Ala1038Val)	c.4234C>T / p.(Gln1412*)
1	63	f	27	34	c.5512C>G / p.(His1838Asp)	c.5882G>A / p.(Gly1961Glu)
1	64	f	12	48	c.5512C>G / p.(His1838Asp)	c.5882G>A / Gly1961Glu)
1	65	m	4	24	c.2588G>C / p.(Gly863Ala)	c.4234C>T / p.(Gln1412*)
1	66	m	6	51	c.3407G>A / p.(Gly1136Glu)	c.5882G>A / p.(Gly1961Glu)
1	67	m	13	15	c.2549A>G / p.(Tyr850Cys)<br>c.5882G>A / p.(Gly1961Glu)	c.5461-10T>C<br>c.5603A>T / p.(Asn1868Ile)
1	68	f	37	48	c.1804C>T / p.(Arg602Trp)	c.5882G>A / p.(Gly1961Glu)
1	69	f	35	35	c.1622T>C / p.(Leu541Pro)<br>c.3113C>T / p.(Ala1038Val)	c.5603A>T / p.(Asn1868Ile)
1	70	f	49	52	c.4066C>T / p.(Gln1356*)<br>c.6334G>C / p.(Val2112Leu)	c.5603A>T / p.(Asn1868Ile)
1	71	f	21	23	c.1622T>C / p.(Leu541Pro)<br>c.3113C>T / p.(Ala1038Val)	c.5603A>T / p.(Asn1868Ile)<br>c.5714+4C>T
2	72	f	49	50	c.455G>A / p.(Arg152Gln) ;<br>c.5603A>T / p.(Asn1868Ile)	c.5461-10T>C<br>c.5603A>T / p.(Asn1868Ile)
2	73	m	40	51	c.3113C>T / p.(Ala1038Val)	c.6118C>T / p.(Arg2040*)
2	74	f	45	50	c.5882G>A / p.(Gly1961Glu)	c.6317G>A / p.(Arg2106His)
2	75	f	70	74	c.4854G>T / p.(Trp1618Cys)	c.5603A>T / p.(Asn1868Ile)
2	76	f	10	13	c.6746C>A / p.(Ala2249Asp)	c.6746C>A / p.(Ala2249Asp)
2	77	m	23	31	c.1622T>C / p.(Leu541Pro)<br>c.3113C>T / p.(Ala1038Val)	c.2588G>C / p.(Gly863Ala)<br>c.5603A>T / p.(Asn1868Ile)
2	78	f	60	61	c.5603 / p.(Asn1868Ile)<br>c.6215G>A / p.(Ser2072Asn)	c.5196+1137G>A<br>c.5329A>T / p.(Met1777Leu)
2	79	f	14	23	c.768G>T / p.(Val256Val)	c.3386G>T / p.(Arg1129Leu)
2	80	f	30	42	c.5196+1483_5313-89del3321bp<br>c.5312+1G>A / p.(Asn1734Glyfs*14)	c.5329A>T / p.(Met1777Leu)
2	81	f	45	55	c.1411G>A / p.(Glu471Lys)	c.1903C>T / p.(Gln635*)
2	82	m	4	13	c.5917delG / p.(Val1973*)	c.5917delG / p.(Val1973*)
2	83	f	39	42	c.4468T>C / p.(Cys1490Arg)<br>c.4685T>C / p.(Ile1562Thr)	c.4458delA / p.(Ser1487Profs*39)
2	84	f	25	45	c.2588G>C / p.(Gly863Ala)<br>c.5603A>T / p.(Asn1868Ile)	c.5461-10T>C<br>c.5603A>T / p.(Asn1868Ile)

2	85	m	25	27	c.1719G>A / p.(Met573Ile)	
2	86	m	37	38	c.470T>A / p.(Leu157*)<br>c.5603A>T / p.(Asn1868Ile)	c.5603A>T / p.(Asn1868Ile)
2	87	f	31	34	c.3195G>A / p.(Glu1399Lys)<br>c.6079C>T / p.(Leu2027Phe)	c.5929G>A / p.(Gly1977Ser)
2	88	f	56	86	c.4462T>C / p.(Cys1488Arg)	
2	89	f	33	42	c.1009T>C / p.(Phe337Leu)<br>c.5603A>T / p.(Asn1868Ile)	c.1937+1G>A
2	90	m	48	51	c.108delT / p.(Leu37Trpfs*3)	
2	91	f	23	53	c.3113C>T / p.(Ala1038Val)	c.6118C>T / p.(Arg2040*)
2	92	f	29	68	c.5882G>A / p.(Gly1961Glu)	c.6229C>T / p.(Arg2077Trp)
2	93	f	39	77	c.1822T>A / p.(Phe608Ile)	c.5603A>T / p.(Asn1868Ile)<br>c.5682G>C / p.(Leu1894Leu);<br>c.4773+48C>T
2	94	m	51	57	c.768G>T / p.(Val256Val)	c.1356+3_1356+4insG
2	95	f	25	43	c.3212C>T / p.(Ser1071Leu)<br>c.66G>A	c.5917delG / p.(Val1973*)
2	96	f	17	69	c.4838delA / p.(Asp1613Alafs*49)	c.5882G>A / p.(Gly1961Glu)
2	97	m	12	22	c.768G>T / p.(Val256Val)	c.5714+5G>A
2	98	m	10	22	c.768G>T / p.(Val256Val)	c.5714+5G>A
2	99	m	50	59	c.2588G>C / p.(Gly863Ala)<br>c.5603A>T / p.(Afn1868Ile)	c.4685T>C / p.(Ile1562Thr)
2	100	f	10	21	c.1622T>C / p.(Leu541Pro)<br>c.3113C>T / p.(Ala1038Val)	c.3212C>T / p.(Ser1071Leu)
2	101	m	08	19	c.1622T>C / p.(Leu541Pro)<br>c.3113C>T / p.(Ala1038Val)	c.3212C>T / p.(Ser1071Leu)
2	102	m	11	11	c.4773+3A>G	c.6118C>T / p.(Arg2040*)
2	103	f	6	28	c.872C>T / p.(Pro291Leu)<br>c.1531C>T / p.(Arg511Cys)	c.872C>T / p.(Pro291Leu)<br>c.1531C>T / p.(Arg511Cys)<br>c.3482G>A / p.(Arg1161His)
2	104	f	14	62	c.3871C>T / p.(Gln1291*)	c.5882G>A / p.(Gly1961Glu)
2	105	f	23	34	c.1654G>A / p.(Val552Ile)<br>c.4771G>A / p.(Gly1591Arg)	c.1622T>C / p.(Leu541Pro)<br>c.3113C>T / p.(Ala1038Val)
2	106	m	68	72	c.1804C>T / p.(Arg602Trp)	c.1928T>G / p.(Val643Gly)
2	107	f	24	37	c.1088T>A / p.(Tyr362*)	c.5603A>T / p.(Asn1868Ile)<br>c.6089G>A / p.(Arg2030Gln)
2	108	m	57	60	c.2894A>G / p.(Asn965Ser)	c.5882G>A / p.(Gly1961Glu)
2	109	f	16	48	c.6089G>A / p.(Arg2030Gln)	c.6545_6580del36bp /<br>p.(Leu2184_Phe2193del)
2	110	f	49	49	c.4527dupC / p.(Gln1513Prof*42)	c.5603A>T / p.(Asn1868Ile)
2	111	f	6	20	c.5714+5G>A	c.5917delG / p.(Val1973*)
2	112	f	51	52	c.3364G>A / p.(Glu1122Lys)<br>c.768G>T / p.(Val256Val)	c.5603A>T / p.(Asn1868Ile)
2	113	m	13	13	c.4793C>A / p.(Ala1598Asp)	c.5460+1G>A
2	114	f	56	58	c.1622T>C / p.(Leu541Pro)<br>c.3113C>T / p.(Ala1038Val)	c.5603A>T / p.(Asn1868Ile)

2	115	f	56	65	c.3289A>T / p.(Arg1097*)	c.5603A>T / p.(Asn1868Ile)
2	116	m	49	62	c.52C>T / p.(Arg18Trp)<br>c.1588G>A / p.(Gly863Ala)<br>c.5603A>T / p.(Asn1868Ile)	c.1715G>A / p.(Arg572Gln) ;<br>c.2828G>A / p.(Arg943Gln)
2	117	m	11	15	c.2041C>T / p.(Arg681*)	c.5381C>A / p.(Ala1794Asp)
2	118	f	30	42	c.45G>A / p.(Trp15*)	c.2401G>A / p.(Ala801Thr)
2	119	m	29	49	c.2588G>C / p.(Gly863Ala)	c.4297G>A / p.(Val1433Ile)
2	120	f	20	44	c.4234C>T / p.(Gln1412*)	c.5882G>A / p.(Gly1961Glu)
2	121	m	7	9	c.1622T>C / p.(Leu541Pro)<br>c.3113C>T / p.(Ala1038Val)	---
2	122	f	25	46	c.2401G>A / p.(Ala801Thr)	c.4234C>T / p.(Gln1412*)
2	123	f	60	68	c.4139C>T / p.(Pro1380Leu)	---
2	124	f	44	51	c.4347G>T / p.(Trp1449Cys)	E12-E13 Deletion
2	125	f	60	66	c. 5603 / p.(Asn1868Ile)<br>c.6215G>A / p.(Ser2072Asn)	c.5196+1137G>A<br>c.5329A>T / p.(Met1777Leu)
2	126	f	67	78	c.4567C>T / p.(Gln1523*)	c.5603A>T / p.(Asn1868Ile)
3	127	f	10	11	c.653G>A / p.(Arg212His)<br>c.1344delG / p.(Met448Ilefs*3)	c.1268G>A / p.(His423Arg)
3	128	f	30	39	c.6746C>A / p.(Ala2249Asp)	c.6746C>A / p.(Ala2249Asp)
3	129	m	21	38	c.5196+1137G>A	E20-22 Deletion
3	130	m	20	47	c.1622T>C / p.(Leu541Pro)<br>c.3113C>T / p.(Ala1038Val)	c.6089G>A / p.(Arg2030Gln)
3	131	f	6	81	c.5603A>T / p.(Asn1868Ile)	c.6419T>G / p.(Leu2140Arg)
3	132	f	30	56	c.1622T>C / p.(Leu541Pro)<br>c.3113C>T / p.(Ala1038Val)	E13 Deletion
3	133	m	46	76	c.6283-2A>G	c.6283-3C>A
3	134	m	40	47	c.5714+5G>A	c.6386+2C>G
3	135	m	12	31	c.4463G>A / p.(Cys1488Tyr)<br>c.6148G>C / p.(Val2050Leu)	c.5714+5G>A
3	136	f	18	40	c.2588G>C / p.(Gly863Ala)<br>c.5603A>T / p.(Asn1868Ile)	c.1622T>C / p.(Leu541Pro)<br>c.3113C>T / p.(Ala1038Val)
3	137	m	6	30	c.4462T>C / p.(Cys1488Arg)	c.4462T>C / p.(Cys1488Arg)
3	138	m	17	41	c.2948C>T / p.(Thr983Ile)<br>c.5603A>T / p.(Afn1868Ile)	c.5714+5G>A
3	139	m	41	51	c.741_744delCGTG /<br>p.(Asn247Lysfs*14)	c.5603A>T / p.(Asn1868Ile)
3	140	w	9	18	c.5549T>C / p.(Leu1850Pro)	c.5509C>A / p.(Pro1837Thr)
3	141	w	9	28	c.5549T>C / p.(Leu1850Pro)	c.5509C>A / p.(Pro1837Thr)
3	142	f	12	40	c.5172G>A / p.(Trp1724*)	c.5172G>A / p.(Trp1724*)
3	143	f	13	37	E20-22 Deletion	c.2588G>C / p.(Gly863Ala)

3	144	m	8	65	c.768G>T / p.(Val256Val)<br>c.1268A>G / p.(His423Arg)	c.1268A>G / p.(His423Arg)<br>c.5603A>T / p.(Asn1868Ile)
3	145	f	9	33	c.214G>A / p.(Gly72Arg)	c.6449G>A / p.(Cys2150Tyr)
3	146	f	10	27	c.1897T>C / p.(Tyr603His)	c.4462T>C / p.(Cys1488Arg)
3	147	m	18	44	c.4793C>A / p.(Ala1598Asp)	c.5460+1G>A
3	148	f	49	64	c.3468C>G / p.(Tyr1156*)	c.5059A>T / p.(Ile1687Phe)
3	149	f	30	61	c.3468C>G / p.(Tyr1156*)	c.5059A>T / p.(Ile1687Phe)
3	150	f	17	23	c.5882G<A / p.(Gly1961Gln)<br>c.5917delG / p.(Val1973*)	c.1622T>C / p.(Leu541Pro) ;<br>c.3113C>T / p.(Ala1038Val)
3	151	f	8	16	c.4234C>T / p.(Gln412Tyr)	c.5461-10T>C
3	152	f	30	62	c.2588G>C / p.(Gly863Ala)<br>c.5603A>T / p.(Asn1868Ile)	c.768G>T / p.(Val256Val)
3	153	f	9	26	c.5917delG / p.(Val1973*)	c.5917delG/ p.(Val1973*)
3	154	m	7	17	c.1622T>C / p.(Leu541Pro)<br>c.3113C>T / p.(Ala1038Val)	c.4234C>T / p.(Glu1412*)
3	155	f	9	14	c.1622T>C / p.(Leu541Pro)<br>c.3113C>T / p.(Ala1038Val)	c.4234C>T / p.(Glu1412*)
3	156	f	5	10	c.5172G>A / p.(Trp1724*)	c.5461-10T>C

f = female; m = male; yrs = years
