# Supplementary material for: Prediction of Function in ABCA4-Related Retinopathy Using Ensemble Machine Learning
Source: J Clin Med. 2020 Jul 29;9(8):2428. doi: 10.3390/jcm9082428 (PMC7463567; doi:10.3390/jcm9082428)
Supplement: Supplementary file 1 [file jcm-09-02428-s001.zip › Supplementary_Table S2 New.pdf]

Supplementary Table S2: Retinal layer thickness measures for each layer and subfield

Supplement to *Prediction of function in ABCA4-related retinopathy using ensemble machine learning*

Layer	ETDRS subfield	Thickness measurements [µm][a]				
		Controls	All patients	Group 1[b]	Group 2[b]	Group 3[b]
IR	Central	104.99±27.14	82.61±23.41	80.79±21.34	86.46±24.54	79.88±24.97
IR	Superior (Intermediate)	188.27±18.57	170.15±20.54	171.45±16.16	170.18±23.09	167.02±24.20
IR	Inferior (Intermediate)	189.87±16.43	163.54±20.09	165.17±12.90	164.53±24.96	157.87±22.87
IR	Nasal (Intermediate)	186.84±13.52	163.26±20.82	162.19±15.80	164.22±24.28	164.05±24.03
IR	Temporal (Intermediate)	175.45±14.79	151.18±20.12	152.24±14.48	151.80±23.81	147.55±23.54
IR	Superior (External)	154.74±13.80	149.83±15.91	151.10±10.03	146.82±14.47	152.32±25.85
IR	Inferior (External)	157.36±14.78	146.78±15.17	148.54±11.00	143.72±16.42	148.22±19.74
IR	Nasal (External)	181.42±12.45	163.70±15.91	166.48±12.15	160.47±17.37	163.03±19.39
IR	Temporal (External)	144.21±13.39	133.66±17.97	136.11±9.99	133.26±19.31	128.59±26.80
ONL	Central	89.30±12.81	24.26±25.14	25.00±25.79	27.87±27.14	15.88±16.36
ONL	Superior (Intermediate)	70.25±8.30	28.12±15.61	35.66±12.30	24.14±16.08	17.55±12.62
ONL	Inferior (Intermediate)	66.04±10.55	25.59±15.61	32.27±13.76	22.10±16.08	16.16±11.36
ONL	Nasal (Intermediate)	73.20±11.43	25.92±16.74	31.72±15.55	22.09±18.28	19.23±11.05
ONL	Temporal (Intermediate)	72.25±8.27	25.77±16.47	33.60±13.97	21.47±17.02	15.12±11.16
ONL	Superior (External)	61.69±7.10	39.09±16.82	49.94±9.23	37.56±14.31	16.20±9.29
ONL	Inferior (External)	53.01±6.93	34.79±13.67	42.07±9.16	35.42±11.68	16.39±7.61
ONL	Nasal (External)	59.28±10.14	35.55±14.57	43.89±10.21	33.74±13.97	19.13±7.67
ONL	Temporal (External)	59.36±6.86	34.20±15.48	43.93±9.03	32.37±13.89	14.54±9.08
ISOS	Central	52.17±5.14	18.59±17.37	22.01±17.13	18.64±19.23	10.40±10.06
ISOS	Superior (Intermediate)	45.57±3.10	25.42±15.48	36.68±9.42	19.48±14.14	9.68±7.58
ISOS	Inferior (Intermediate)	44.97±2.71	22.14±14.96	32.04±11.76	16.95±13.44	8.23±5.52
ISOS	Nasal (Intermediate)	47.10±3.42	21.83±14.94	31.26±12.69	16.71±13.17	8.89±5.89
ISOS	Temporal (Intermediate)	46.25±3.14	22.23±15.84	33.27±12.25	15.82±13.91	7.84±4.83
ISOS	Superior (External)	43.14±2.28	34.34±15.03	43.62±5.56	33.96±13.06	13.10±11.54
ISOS	Inferior (External)	42.17±1.99	33.48±14.04	42.17±5.29	33.67±10.88	12.56±11.17
ISOS	Nasal (External)	44.07±2.91	33.00±13.97	42.27±5.83	30.87±12.49	14.97±10.61
ISOS	Temporal (External)	43.87±2.43	31.84±15.77	42.86±6.02	29.44±13.95	10.15±9.07
RPE	Central	38.65±3.50	30.64±7.55	33.33±7.09	30.53±6.99	24.44±5.65
RPE	Superior (Intermediate)	36.32±3.00	33.17±6.70	37.05±4.71	31.91±5.78	26.29±5.81
RPE	Inferior (Intermediate)	36.45±2.58	31.73±7.07	35.88±5.31	30.24±6.22	24.63±5.19
RPE	Nasal (Intermediate)	37.20±2.65	31.69±6.84	35.47±5.44	30.46±6.05	25.01±5.04
RPE	Temporal (Intermediate)	36.02±2.33	31.18±6.69	35.29±4.98	29.55±5.74	24.44±4.84
RPE	Superior (External)	36.41±2.68	36.13±6.17	39.59±3.54	36.35±5.15	27.54±4.17
RPE	Inferior (External)	35.92±2.14	35.06±5.55	38.02±3.32	35.21±4.34	27.78±5.09
RPE	Nasal (External)	35.81±2.51	35.09±5.46	38.30±3.51	34.67±4.59	28.28±4.05
RPE	Temporal (External)	34.71±2.09	34.29±6.36	38.14±3.65	33.72±4.96	26.25±5.76

[a] Values indicate the mean ± standard deviation.

[b] Groups are classified according to full-field electroretinogram classification.[15]

ONL: outer nuclear layer, IR: inner retina, ISOS: photoreceptor inner and outer segments, RPE: retinal pigment epithelium.
