# Supplementary material for: Prediction of Function in ABCA4-Related Retinopathy Using Ensemble Machine Learning
Source: J Clin Med. 2020 Jul 29;9(8):2428. doi: 10.3390/jcm9082428 (PMC7463567; doi:10.3390/jcm9082428)
Supplement: Supplementary file 1 [file jcm-09-02428-s001.zip › Supplementary_Figure S1.pdf]

Supplementary Figure S1: Visualization of the nested cross-validation

Supplement to *Prediction of function in ABCA4-related retinopathy using ensemble machine learning*

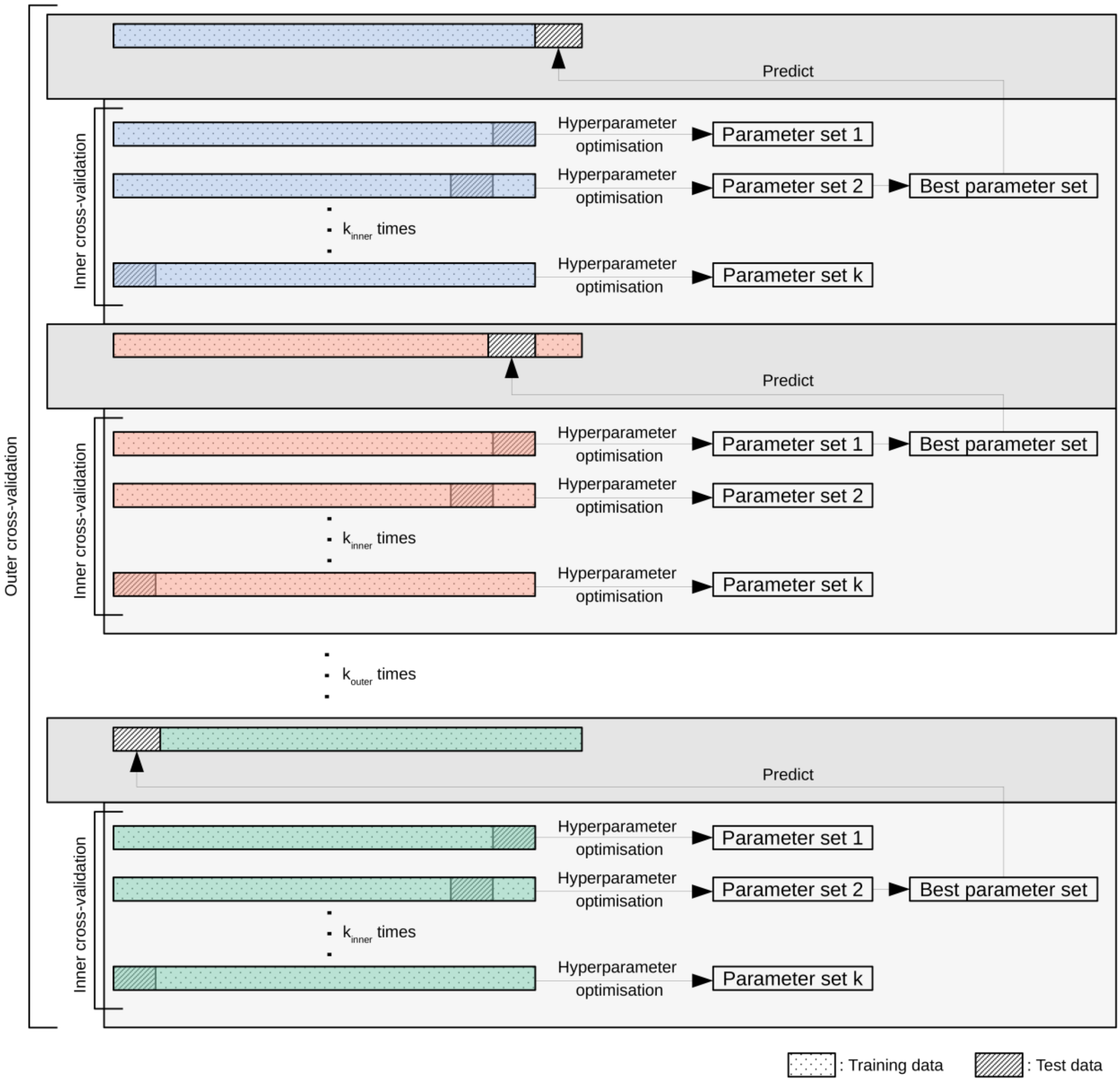

To avoid an optimization bias, the evaluation of the model performance was separated from the hyperparameter tuning by using nested cross-validation. For this, two parameters k_{outer} and k_{inner} were chosen. For the outer loop, $(100/k_{outer})*(k_{outer}-1)$ % of data was chosen to be the outer training set and the remaining $(100/k_{outer})$ % were used as the outer test data set. Then, the previously chosen outer training data was iterated over in k_{inner} steps as described for k_{outer} creating an inner test and training data set. The models were then trained on the inner training set predicting the inner test set. For each k_{outer} fold, the hyperparameter set that achieved the best performance on the inner test set was used to predict the outer test set. This was repeated for all k_{outer} folds. The order of data was randomized before conducting the nested cross-validation to exclude a sorting bias in the data.
