# Supplementary material for: Prediction of Function in ABCA4-Related Retinopathy Using Ensemble Machine Learning
Source: J Clin Med. 2020 Jul 29;9(8):2428. doi: 10.3390/jcm9082428 (PMC7463567; doi:10.3390/jcm9082428)
Supplement: Supplementary file 1 [file jcm-09-02428-s001.zip › Supplementary_Figure S2.pdf]

Supplementary Figure S2: Cohort characteristics of included participants

Supplement to *Prediction of function in ABCA4-related retinopathy using ensemble machine learning*

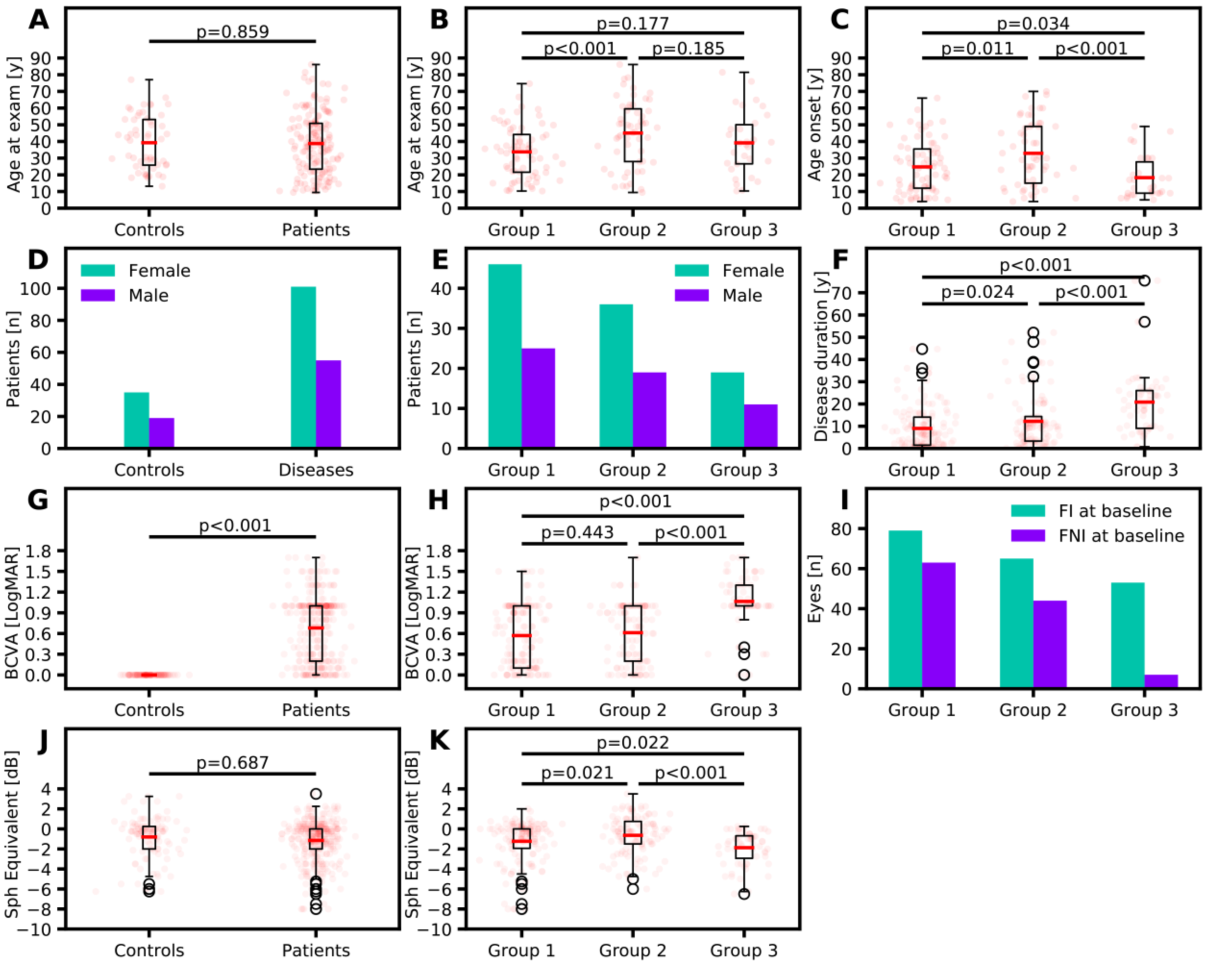

Individual values (red circles) or absolute numbers (bars) of controls and patients (first column) and/or the three disease groups based on full-field ERG classification (second and third column) are shown.15 (A) There was no significant difference in the distribution of age between controls and patients. (B) Patients assigned to group 1 were overall slightly younger and group 2 patients had a trend to an older age. (C) The latter revealed also a higher age of onset, while group 3 patients suffered from first symptoms at the youngest age. (D) The distribution of sex was not significantly different between controls and patients (p=0.876), (E) similar to the disease groups (p=0.981). (F) The disease duration increased from group 1 to group 3 patients. (G) The spherical equivalent (Sph Equivalent) was similar between controls and patients, (H) while group 3 eyes revealed significant higher myopia. (I) Concerning the foveal status, group 3 eyes showed a significantly higher proportion of foveal involving (FI) RPE atrophy (p=0.007) and only rare cases of foveal non-involvement (FNI). (J) Patients revealed significantly impaired best corrected visual acuity (BCVA; demonstrated in the logarithm of the minimum angle of resolution, LogMAR), (K) most expressed in group 3 eyes. A-F are based on patient-level data, G-K on eye-level data. The boxplots demonstrate the mean (red line) and quartiles (box, q1-q3; error bars, q0-q4; black circles, outliners). Comparisons are based in Welch's t-test (for interval scaled data) and chi-squared test (for frequencies).
