# Supplementary material for: Prediction of Function in ABCA4-Related Retinopathy Using Ensemble Machine Learning
Source: J Clin Med. 2020 Jul 29;9(8):2428. doi: 10.3390/jcm9082428 (PMC7463567; doi:10.3390/jcm9082428)
Supplement: Supplementary file 1 [file jcm-09-02428-s001.zip › Supplementary_Figure S3 New.pdf]

Supplementary Figure S3: Retinal layer thickness measurements

Supplement to *Prediction of function in ABCA4-related retinopathy using ensemble machine learning*

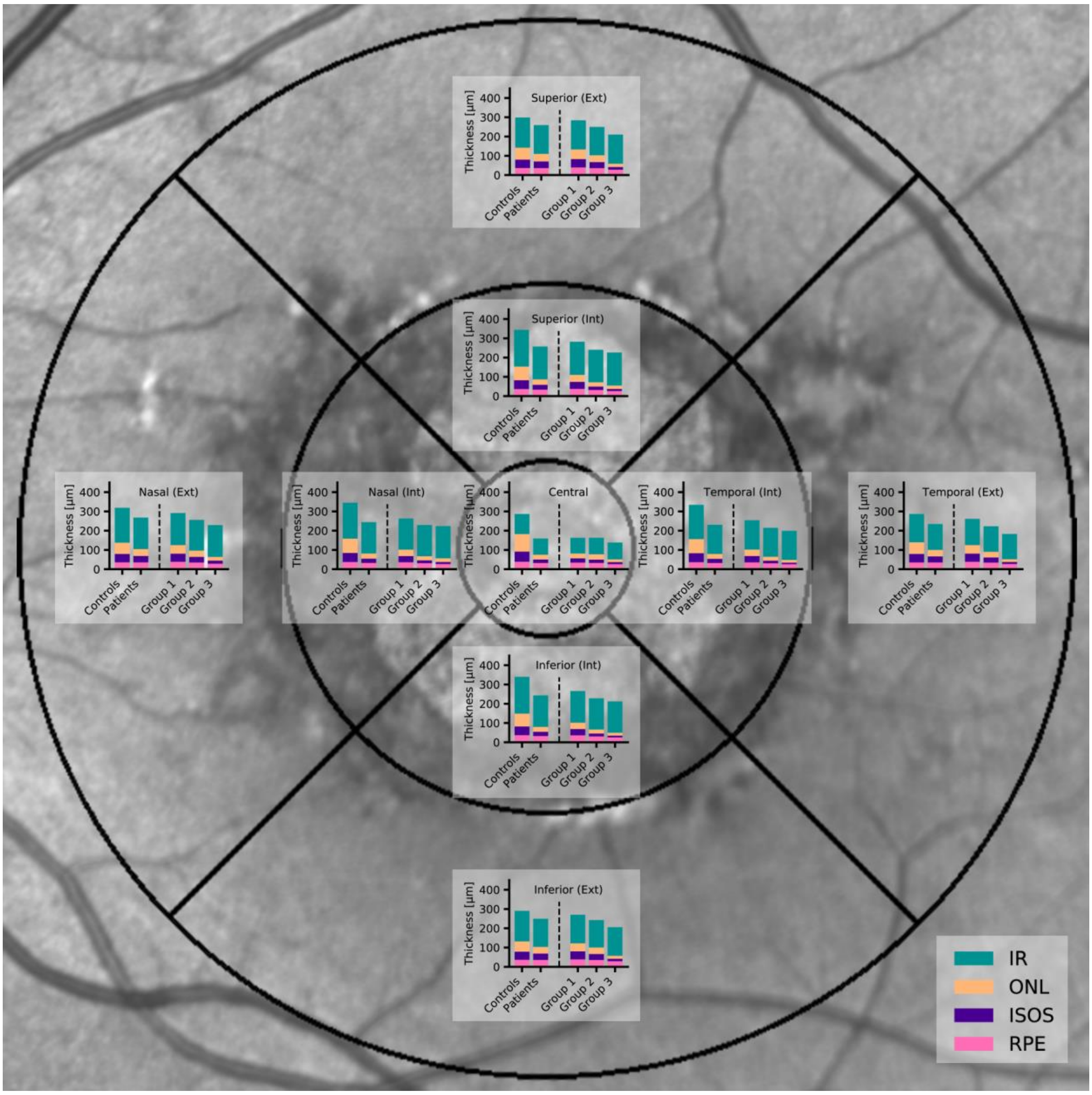

Overlaid onto an infrared reflectance image and the ETDRS grid used for image analysis, bar plots show the mean retinal layer thickness measurements in each of the nine ETDRS subfields, separated for controls, all patients, and the three patient groups. Compared to controls, patients' eyes revealed reduced retinal thickness in each subfield. This finding mainly derived from thinning of outer nuclear layer (ONL, yellow) and photoreceptor inner and outer segment layers (ISOS, purple), while inner retina layers (IR, green) and retinal pigment epithelium layer (RPE, pink) overall did not show such a finding. The only exception were eyes assigned to group 3 that consistently revealed thinnest RPE measures in all subfields. In the central subfield, the retinal thinning was expressed in eyes assigned to all groups of patients, while in the more eccentrically subfields it was increasingly expressed from group 1 over group 2 to group 3 eyes. Exact numbers can be found in Supplementary Table S2. Ext: external, Int: intermediate.
