# Supplementary material for: Prediction of Function in ABCA4-Related Retinopathy Using Ensemble Machine Learning
Source: J Clin Med. 2020 Jul 29;9(8):2428. doi: 10.3390/jcm9082428 (PMC7463567; doi:10.3390/jcm9082428)
Supplement: Supplementary file 1 [file jcm-09-02428-s001.zip › Supplementary_Figure S4.pdf]

Supplementary Figure S4: Directional differences in retinal thickness

Supplement to *Prediction of function in ABCA4-related retinopathy using ensemble machine learning*

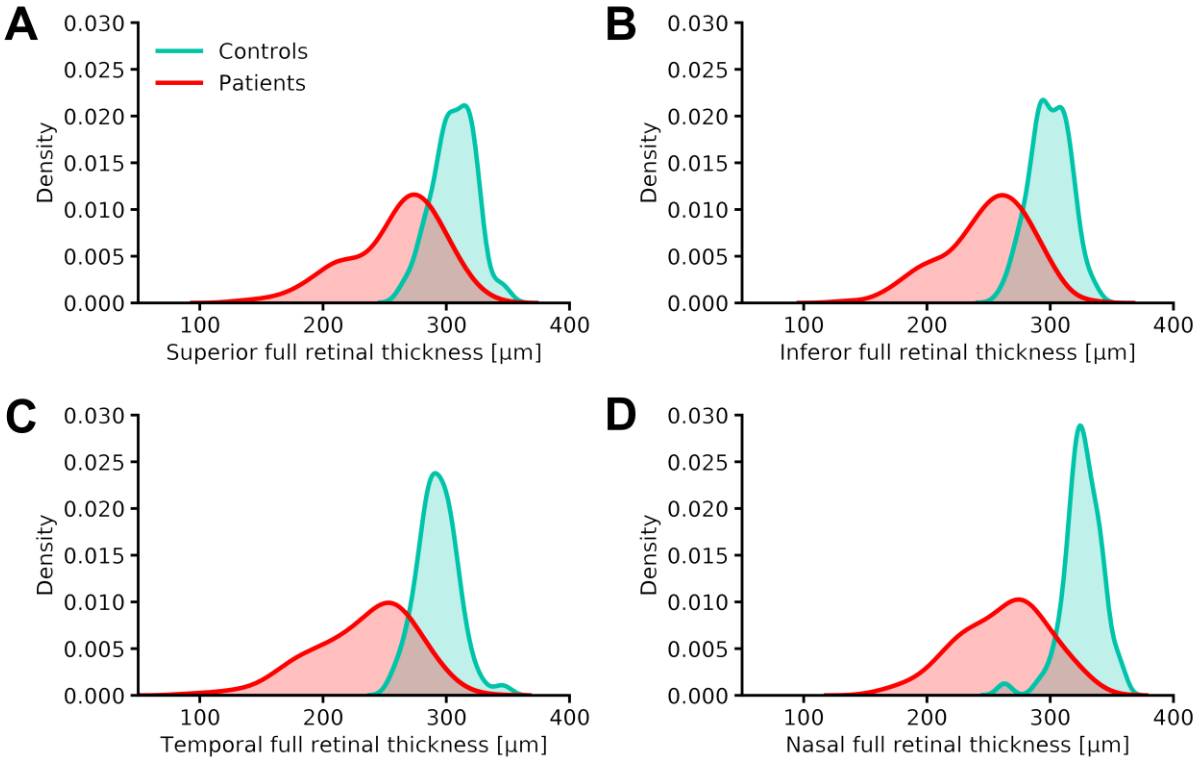

The density plots show the distributions of the full retinal thickness of patients (red) and controls (cyan). In all directions, patients revealed thinner retinal thickness ($P<0.001$), which was most and least expressed in the nasal (D) and superior (A) ETDRS subfields, respectively. Of note, the nasal full retina thickness also revealed the overall highest values.
