# Supplementary material for: Prediction of Function in ABCA4-Related Retinopathy Using Ensemble Machine Learning
Source: J Clin Med. 2020 Jul 29;9(8):2428. doi: 10.3390/jcm9082428 (PMC7463567; doi:10.3390/jcm9082428)
Supplement: Supplementary file 1 [file jcm-09-02428-s001.zip › Supplementary_Figure S5.pdf]

Supplementary Figure S5: Feature importance for the machine learning algorithms

Supplement to *Prediction of function in ABCA4-related retinopathy using ensemble machine learning*

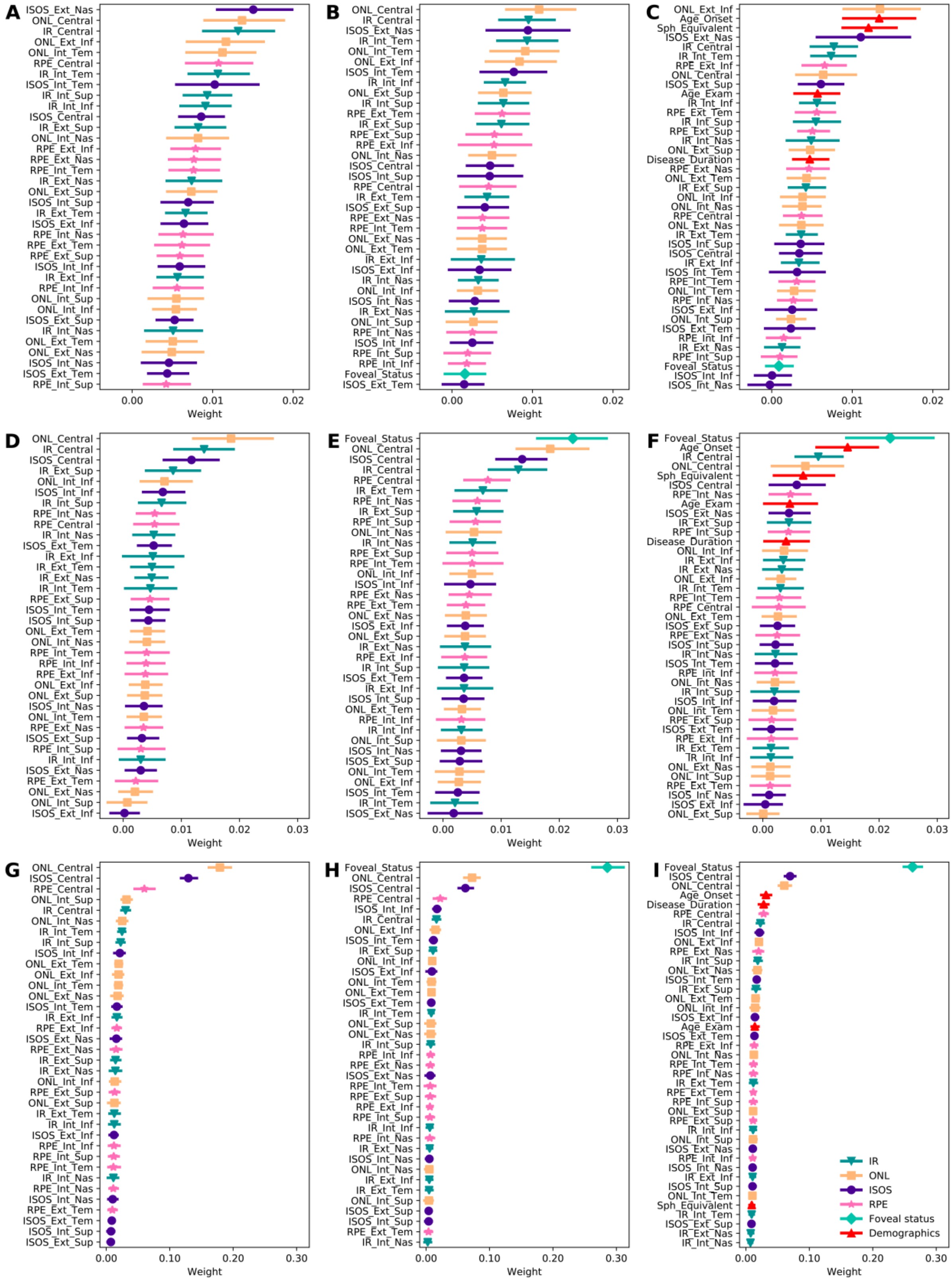

The panels display the mean weights (symbol) and the 95 % confidence interval (error bars) of the permutation importance for prediction of full-field electroretinogram (ERG) categories (first row), grades of visual impairment (second row), and best corrected visual acuity (BCVA) values (third row). The first, second, and third column represent the feature sets A, B, and C, respectively. For all predictions, the thickness measures of photoreceptor inner and outer segments (ISOS, purple dot), outer nuclear layer (ONL, yellow square), and inner retina (IR, green inverted triangle) ranked among the most significant impact. Thickness measures of the retinal pigment epithelium (RPE, pink arrowhead) were less commonly found within the first quarter. While all eccentricities were present in the highest ranked features for ERG predictions (A-C), the models for visual impairment and BCVA prediction focused more on measurements in the central subfield (D-I). For the latter models, the foveal status (cyan diamond) carried the most important impact (E, F, H and I), in contrast to the ERG predictions, where the information of foveal status was less important (B, C). Age of onset (red triangle) was consistently found in the group of factors with highest weights, while the other demographic factors (red triangle) ranked differently depending on the model (C, F, I). Ext: external, Inf: inferior, Int: intermediate, Nas: nasal, Sph: spherical, Sup: superior, Tem: temporal.
