# Supplementary material for: Prediction of Function in ABCA4-Related Retinopathy Using Ensemble Machine Learning
Source: J Clin Med. 2020 Jul 29;9(8):2428. doi: 10.3390/jcm9082428 (PMC7463567; doi:10.3390/jcm9082428)
Supplement: Supplementary file 1 [file jcm-09-02428-s001.zip › Supplementary_Figure S6.pdf]

Supplementary Figure S6: Deviation of the best corrected visual acuity (BCVA) predictions

Supplement to *Prediction of function in ABCA4-related retinopathy using ensemble machine learning*

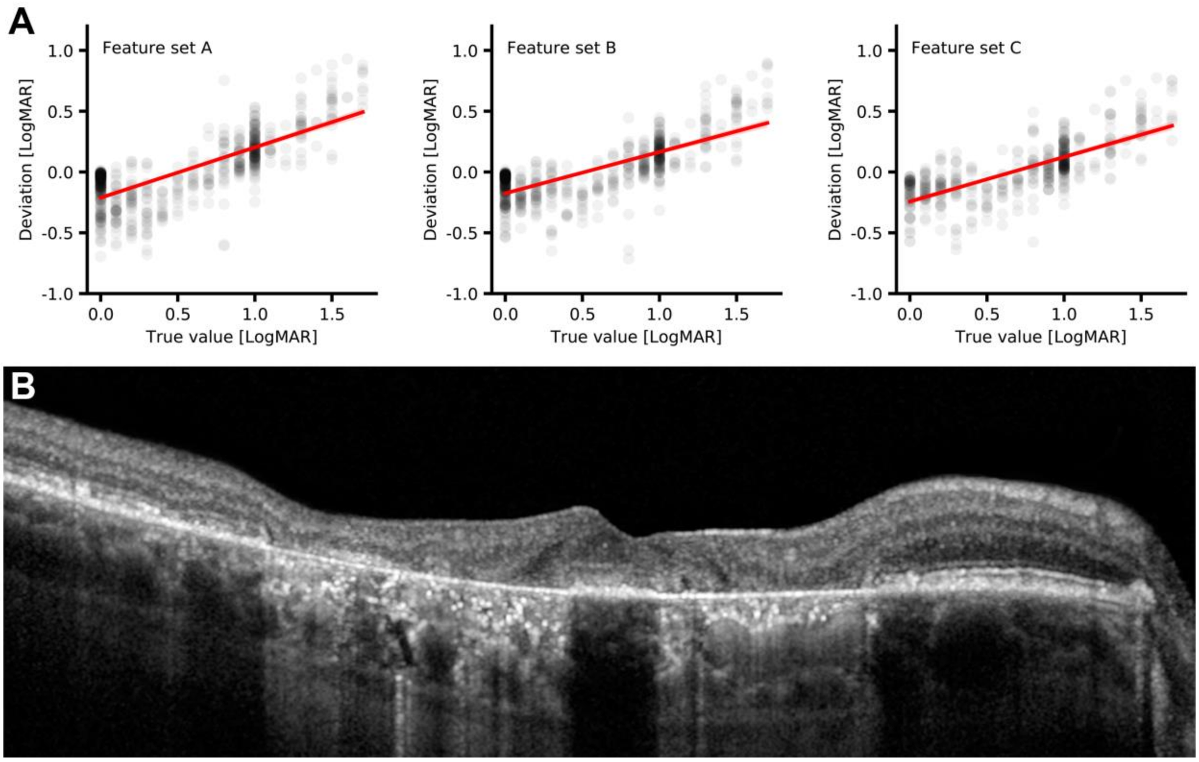

(A) The deviation of machine learning based predictions dependent on the true BCVA values separated for both feature sets are shown. There was a high proportion of eyes with BCVA of 0.0 and 1.0 Logarithm of the Minimum Angle of Resolution (LogMAR). As the model focused more on common values to correctly predict the maximum number of values, deviation of the predictions increased towards the extreme BCVA values. The under- and overestimation by the machine learning based predictions for good and severely impaired visual acuity was slightly more pronounced in feature set A and C. (B) A central horizontal optical coherence tomography B-scan of an exemplary eye showing a potential reason for cases with high deviation is demonstrated. Here the fovea is partly affected by atrophy, partly preserved. In these cases, eyes were assigned to foveal involvement that would lead the model to predict distinctly impaired BCVA, while in fact having better BCVA.
